# Symptom control after different duration of triptorelin treatment following conservative surgery for deep infiltrating endometriosis: Post-hoc analysis of a multicentre, prospective, real-world study

**DOI:** 10.1097/MD.0000000000026753

**Published:** 2021-07-30

**Authors:** Wenting Sun, Keqin Hua, Li Hong, Juxin Zhang, Min Hao, Jianliu Wang, Jun Zhang, Valerie Perrot, Hongbo Li, Xinmei Zhang

**Affiliations:** a Gynaecology Department, Women's Hospital, School of Medicine, Zhejiang University, Hangzhou, China; b Gynaecology Department, Obstetrics & Gynecology hospital of Fudan University, Shanghai, China; c Gynaecology Department, Remin Hospital of Medical Faculty, Wuhan University, Wuhan, Hubei Province, China; d Gynaecology Department, Henan Provincial People's Hospital, Zhengzhou, Henan Province, China; e Gynaecology Department, The Second Hospital of Shanxi University, Taiyuan, Shanxi Province, China; f Gynaecology Department, People's Hospital, Peking University, Beijing, China; g Gynaecology Department, Beijing Anzhen Hospital, Capital Medical University, Beijing, China; h Clinical Statistics department, Ipsen, Boulogne-Billancourt, France; i Medical Affairs department, Ipsen (Beijing) Pharmaceutical Science and Technology Development Co., Ltd, Beijing, China.

**Keywords:** deep infiltrating endometriosis, post-operative, treatment duration, triptorelin

## Abstract

Triptorelin has been used after surgery in deep infiltrating endometriosis. This post-hoc analysis aimed to evaluate symptom control between patients receiving 1-3 triptorelin injections and those receiving 4–6 injections within 24 months of conservative surgery for deep infiltrating endometriosis, in the real-world.

Included patients were divided into two groups (received up to 3 months injections in group A, 4–6 injections in group B) based on the numbers of triptorelin (Diphereline, 3.75 mg intramuscular injection once every 28 days for up to 24 weeks) administration. Evolution in score of pain intensity at 3, 6, 9, 12, 18, and 24 months after primary triptorelin administration and symptom improvement/recurrence rates between two groups were compared. Symptoms of pain intensity were assessed using a visual analogue scale (VAS) with a range from 0 to 10 cm. An improvement in symptoms was defined as a reduction of at least 3 cm or 3 units from pre-surgery levels.

156 patients in group A and 228 in group B. Pain symptom score (mean ± standard deviation) diminished to a nadir at 3-months for group A and 6-months for group B; at 6-months nadir scores were significantly lower in group B (0.9 ± 1.7 vs 0.4 ± 1.2 respectively, *P* = .002). No significant difference for pain symptom scores between both groups at 24-months (*P* = .269). The 6-month and 24-month cumulative improvement rates of pain (80.6% vs 89.8%, *P* = .014 and 82.6% vs 90.7%, *P* = .025) and gastro-intestinal symptoms (61.0% vs 80.8%, *P* = .022 and 61.0% vs 83.3%, *P* = .008) were significantly higher in group B, whereas there was no significant difference in rates of menstrual disorders and urinary symptoms. There is no significant difference for 12-months and 24-months cumulative recurrence rates of total symptoms between both groups (11.3% vs 13.8%, *P* = .568 and 16.1% vs 26.0%, *P* = .094).

In women with deep infiltrating endometriosis, longer treatment with triptorelin following conservative surgery was associated with a decrease in symptom intensity and greater improvement of pain symptoms in the short-term and greater improvement of gastro-intestinal symptoms in the long-term.

Trial registration number: ClinicalTrials.gov, NCT01942369.

## 1 Introduction

Deep infiltrating endometriosis (DIE) as the most debilitating form of endometriosis is estimated to affect more than 20% of women with endometriosis.^[1,2]^ It is defined by the infiltration by endometriotic tissue beneath the peritoneum of more than 5 mm with symptoms of severe pain and infertility.^[3,4]^ DIE is responsible for various symptoms such as chronic pelvic pain, coital pain, dysmenorrhea, menstrual urinary and intestinal symptoms.^[3]^ Moreover, DIE affects several locations including uterosacral ligament, bladder and ureter, intestine, vaginal rectovaginal septum, vagina, etc.^[3]^ It greatly reduces the quality of life of patients, meanwhile it increases the difficulty of treatment and increases the financial burden of their families.^[5]^

Surgery is often required for patients with DIE aiming at excising affected tissues to achieve symptom relief and restore fertility.^[6]^ Compared with radical surgical operation, conservative surgery might have a reduction of trauma and complications, so conservative surgery is also considered the preferable option aiming at complete disease excision. Conservative surgery can be appropriate for many patients with DIE and more patients may benefit from rectal sparing procedures.^[6–9]^ However, the rate of symptom recurrence after conservative surgical approach is very high, long-term care and medical therapy is always warranted.^[2]^

Pharmacological therapy combined with conservative surgical treatment have been proved to achieve the best therapeutic effects.^[5]^ Medical treatments include combined oral contraceptive pills, danazol, gestrinone, progestogen, and gonadotropin-releasing hormone agonists (GnRH-a).^[10–12]^ It has been proved that GnRH-a have an important role in the treatment of endometriosis after conservative surgery with the aim to help relieve pain and reduce the risk of recurrence.^[5,13–15]^ Currently, it is widely used for the therapy of endometriosis.^[16]^ Moreover, GnRH-a therapy following conservative surgery is appreciated to be a good choice in the long-term care of patients with DIE. However, the use of GnRH-a is usually limited to 6 months due to risk of bone loss for longer prescription.^[13]^

Triptorelin (D-Trp6-LHRH) is one of the most commonly used GnRH-a and can improve post-operative pain symptoms, such as dysmenorrhoea, dyspareunia and pelvic pain.^[17,18]^ A prospective observational study performed in China investigated the effects of triptorelin in the treatment of patients with endometriosis, using a 6-week versus 4-week triptorelin drug regimen.^[19]^ The study suggested that similar efficacy and symptom recurrence were achieved from both therapeutic regimens. However, the single-centre study was with a small sample and a narrow population. The duration and efficacy of post-operative triptorelin therapy remains the subject of debate in China.

This was a post-hoc analysis of a multicentre, prospective, real-world study whose publication were in press. The primary objectives were to evaluate and compare the evolution of symptom score and improvement rates by the number of triptorelin injections within 24 months after surgery for DIE. The secondary objectives were to compare symptom recurrence and pain-free intervals.

## 2 Materials and methods

The study was approved by the Institutional Review Boards of the hospitals in which it was performed and the Institutional Review Boards of Women's Hospital, School of Medicine, Zhejiang University approved the study. It was registered at www.clinicaltrials.gov, number NCT01942369.

### 2.1 Participants and clinical characteristics

A prospective real-world study was performed in 18 tertiary hospitals in China. Premenopausal Chinese women aged ≥18 years old with a diagnosis of DIE who had undergone surgery prior to triptorelin (Diphereline, Ipsen Pharma Biotec, Paris, France) 3.75 mg intramuscular injections (every 28 days for ≤24 weeks; ≤6 injections) therapy at the participating centres and who were mentally and physically able to describe their symptoms and answer questions between September 2013 to July 2016 were included. Patients who were pregnant or lactating, who might reach menopause within 3 years after surgery, with a history of allergic reaction of triptorelin or one of the excipients, with a history of treatment of other drugs within 3 months and GnRH-a therapy within 6 months prior to the study, who were potentially non-compliant or unsuitable for the study for other reasons were excluded.

In the post-hoc analysis, patients were divided into two groups based on the duration of triptorelin administration, patients received 1 to 3 injections in group A, 4 to 6 injections in group B. Information of pain, menstrual disorders, gastro-intestinal and urinary discomfort were focused on and collected during 3-monthly (first-year follow-up) and 6-monthly (second-year follow-up) routine post-operative hospital visits. All participants were followed for a period of 24 months after surgery.

Baseline characteristics including age, body mass index, DIE lesion, history of surgical and hormonal therapy, and symptoms (i.e. pain, menstrual disorders, gastro-intestinal and urinary symptom) intensity between two groups were collected and compared.

The study was approved by the respective Ethical Committees of all participating sites. An informed consent was signed by all eligible participants.

### 2.2 Outcomes of interest

Primary outcomes of interest were comparisons between the two groups in evolution in score of pain intensity (visual analogue scale) and cumulative symptom improvement rates, at 3, 6, 9, 12, 18, and 24 months after primary triptorelin therapy. The definition of symptom improvement was a reduction of at least 3 cm or 3 units from pre-surgery.

Secondary outcomes of interest were to compare symptom recurrence and time to relapse of pain between the two groups. Symptom recurrence was defined as an increase of more than 3 cm or 3 units compared to the lowest previous score. Pain-free interval is the time elapsed from date of disappearance of symptoms (at month 3 or month 6) up to the first occurrence of pelvic pain, dysmenorrhoea, pain at time of ovulation or dyspareunia (visual analogue scale > 3). Patients without occurrence of pain were censored at the date of the last study visit.

Duration of triptorelin treatment was the interval between first and last dose. The predictive factors of triptorelin duration were explored.

### 2.3 Statistical analysis

Continuous variables were expressed as mean ± standard deviation and compared with F test or analysis of variance. Categorical variables are expressed as frequency/proportions and compared using a χ^2^ test or Fisher's exact test or Wilcoxon rank sum test for ordinal variables. The evolution of symptom pain intensity was compared by F test or analysis of variance. The cumulative improvement and recurrence rates between the two groups were compared by univariate logistic regression. The time to relapse of pain was assessed and survival curves in the two groups were drawn using Kaplan-Meier method and compared by Log-rank test. Univariate and multivariate Cox regression models were conducted to identify the predictive factors of triptorelin duration among the demographics and clinical characteristics at baseline. Variables included in the multivariate analysis were those with a *P*-value < .2 in the univariate analysis. Statistical analysis was performed using the software of SAS version 9.21 (SAS Institute, Cary, NC). *P* < .05 was considered statistically significant.

## 3 Results

### 3.1 Baseline characteristics

**Figure 1:**
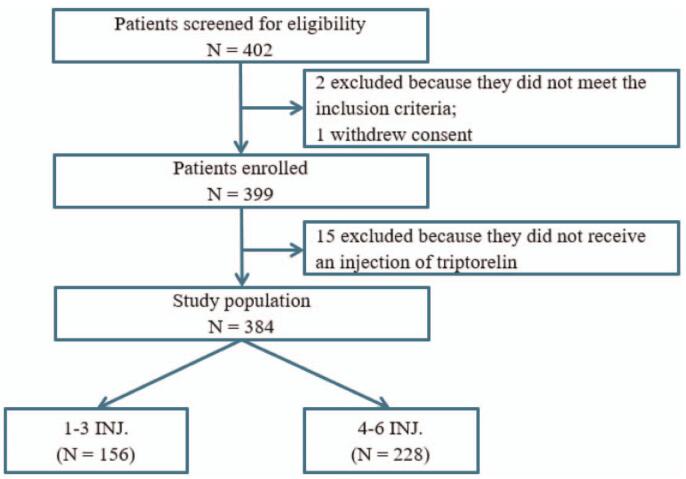
Flow diagram of study population.

In total, 402 patients were screened for eligibility, 2 excluded because of not meeting the inclusion criteria and 1 withdrew consent, and 399 were enrolled. Of them, 15 patients who did not receive an injection of triptorelin were excluded, therefore, 384 (96.2%) women who received triptorelin therapy with diagnosis of DIE were included in the final analysis (Fig. 1). Among them, 156 (40.6%) patients were in group A with a mean age of 32.8 ± 5.7 years and 228 (59.4%) in group B with a mean age of 33.7 ± 6.5 years (*P* = .162).

Table 1 shows demographic and clinical characteristics of participants who had up to 3 triptorelin injections versus those who had 4–6 injections. Baseline characteristics were not significantly different between the two groups except for location of DIE lesions; fewer patients in group A had intestinal (49.4% vs 59.6%, *P* = .048) or vaginal lesions (5.8% vs 12.3%, *P* = .036).

**Table 1:** Demographics and baseline characteristics of study population.

	Group A		Group B		
Baseline characteristics	N	mean ± SD / n (%)	N	mean ± SD / n (%)	*P*-value
Age	156	32.8 ± 5.7	228	33.7 ± 6.5	.162 ^*α*^
BMI	153	21.1 ± 2.7	226	21.0 ± 2.9	.733 ^*α*^
Age at first endometriosis symptoms	143	30.1 ± 6.9	184	30.4 ± 7.5	.723 ^*α*^
Age when first medical attention sought	143	30.9 ± 6.1	184	31.7 ± 6.5	.264 ^*α*^
Age at first endometriosis surgical diagnosis	24	28.7 ± 3.2	56	30.4 ± 5.9	.175 ^*α*^
Ever had a hormonal treatment for endometriosis	156	23 (14.7)	228	40 (17.5)	.487 ^*β*^
Oral contraceptive pills treatment duration	5	4.6 ± 1.9	5	25.8 ± 32.4	.182 ^α^
Gn-RH agonists treatment duration	3	4.3 ± 1.5	15	3.9 ± 2.0	.704 ^α^
Traditional Chinese medication treatment duration	18	8.0 ± 11.5	20	12.9 ± 17.9	.328 ^α^
Ovarian endometrioma	156	149 (95.5)	228	208 (91.2)	.154 ^γ^
Main DIE lesions	156		228		
Ureter		6 (3.8)		4 (1.8)	.328 ^γ^
Intestine		77 (49.4)		136 (59.6)	.048 ^γ^
Bladder		5 (3.2)		3 (1.3)	.279 ^γ^
Associated DIE lesions	156		228		
Vagina		9 (5.8)		28 (12.3)	.036 ^γ^
Left uterosacral ligament		20 (12.8)		22 (9.6)	.405 ^γ^
Right uterosacral ligament		12 (7.7)		19 (8.3)	.852 ^γ^
Bilateral uterosacral ligament		70 (44.9)		124 (54.4)	.077 ^γ^

**Figure 2:**
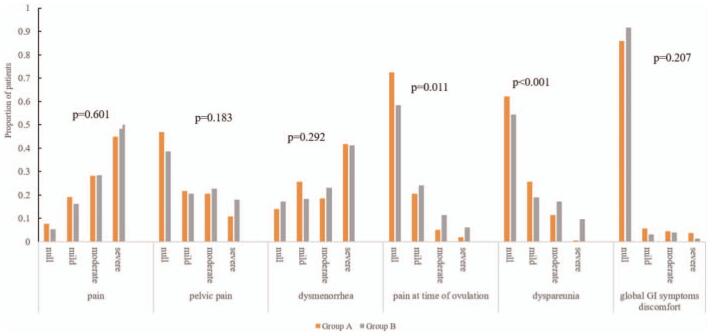
Proportion of patients with different symptom intensity pre-surgery.

At baseline, the proportions of women reporting pain symptoms in group A and group B were respectively null (7.7%, 5.3%), mild (19.2%, 16.2%), moderate (28.2%, 28.5%) and severe (44.9%, 50.0%), the difference between two groups was without significance (*P* = .601). The proportions of pain at time of ovulation in both groups were null (72.4%, 58.3%), mild (20.5%, 24.1%), moderate (5.1%, 11.4%) and severe (1.9%, 6.1%), the difference was significant (*P* = .011). The proportions of dyspareunia in two groups were null (62.2%, 54.4%), mild (25.6%, 18.9%), moderate (11.5%, 17.1%) and severe (0.6%, 9.6%) with statistical significance (*P* < .001) (Fig. 2).

The proportion of previously surgically diagnosed with endometriosis in group A was significantly lower than that in group B (15.4% vs 24.6%, *P* = .030). The differences of others surgical history were not statistically significant (Table 2).

**Table 2:** Surgical history of study population.

	Group A		Group B		
Baseline characteristics	N	n (%)	N	n (%)	*P*-value
Previously surgically diagnosed with endometriosis	156	24 (15.4)	228	56 (24.6)	.030 ^*β*^
Ever operated for endometriosis (other than DIE)	24	10 (41.7)	56	33 (58.9)	.221 ^*β*^
No. of previous Non DIE-endometriosis surgery-Laparoscopy	10		33		.522 ^γ^
0		4 (40.0)		10 (30.3)	
1		6 (60.0)		22 (66.7)	
2		0 (0.0)		1 (3.0)	
No. of previous Non DIE-endometriosis surgery-Lower midline incision	10		33		.905 ^γ^
0		6 (60.0)		21 (63.6)	
1		4 (40.0)		11 (33.3)	
2		0 (0.0)		1 (3.0)	
No. of previous Non DIE-endometriosis surgery-Pfannenstiel incision	10		33		.078^γ^
0		9 (90.0)		33 (100.0)	
1		1 (10.0)		0 (0.0)	
Ever operated for DIE	24	1 (4.2)	56	3 (5.4)	1.000 ^*β*^
No. of previous DIE surgery-Laparoscopy	1		3		.617^γ^
0		1 (100.0)		1 (33.3)	
1		0 (0.0)		2 (66.7)	
No. of previous DIE surgery-Lower midline incision	1		3		.617^γ^
0		0 (0.0)		2 (66.7)	
1		1 (100.0)		1 (33.3)	
Surgical procedure	156		228		.706 ^γ^
Laparoscopy		151 (96.8)		218 (95.6)	
Laparoscopy + Laparotomy		0 (0.0)		2 (0.9)	
Laparotomy		5 (3.2)		8 (3.5)	

### 3.2 Outcomes of interest

**Figure 3:**
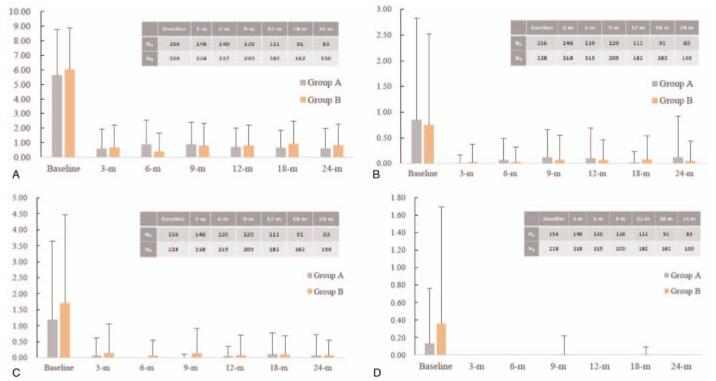
Summary of symptoms scores for study population in 24 months.

All symptom scores decreased significantly between baseline and 3 months and remained stable until 24 months (Fig. 3). Pain symptom score (mean ±  standard deviation) decreased from baseline to nadir (5.6 ± 3.2 vs 0.6 ± 1.3, *P* < .001) at 3-months in group A, whereas the score decreased to nadir (6.0 ± 2.9 vs 0.4 ± 1.2, *P* < .001) at 6-months in group B; at 6-months nadir scores were significantly lower in group B (0.9 ± 1.7 vs 0.4 ± 1.2, *P* = .002). No significant difference between both groups at 24 months (0.6 ± 1.4 vs 0.8 ± 1.5, *P* = .269). Scores of menstrual disorders decreased from baseline to nadir at 3-months in both of group A (0.9 ± 2.0 vs 0.0 ± 0.2, *P* < .001) and group B (0.8 ± 1.8 vs 0.0 ± 0.4, *P* < .001). The 24-months scores were no statistical significance between two groups (0.1 ± 0.8 vs 0.1 ± 0.4, *P* = .395). Gastrointestinal symptom scores decreased from baseline to nadir at 6-months in both of group A (1.2 ± 2.5 vs 0.0 ± 0.0, *P* < .001) and group B (1.7 ± 2.8 vs 0.1 ± 0.5, *P* < .001). The 24-months scores were without statistical significance (0.1 ± 0.7 vs 0.1 ± 0.5, *P* = .898).

The 6-month improvement rate of pain symptoms in group B was significantly higher than that in group A (80.8% vs 90.2%, *P* = .015). Likewise, the 18-month and 24-month improvement rates of gastro-intestinal symptoms in group B were significantly higher than those in group A (55.6% vs 80.4%, *P* = .023 and 59.0% vs 78.2%, *P* = .032). Moreover, the cumulative symptom improvement rates of 6 follow-up visits in group B were all higher than those in group A (Table 3). The 6-month and 24-month cumulative rates of symptoms were significantly higher in group B than those in group A for pain (80.6% vs 89.8%, *P* = .014 and 82.6% vs 90.7%, *P* = .025) and gastro-intestinal symptoms (61.0% vs 80.8%, *P* = .022 and 61.0% vs 83.3%, *P* = .008), whereas there was no significant difference in rates of menstrual disorders (64.5% vs 78.6%, *P* = .187 and 64.5% vs 78.6%, *P* = .187) and urinary symptoms (50.0% vs 61.9%, *P* = .562 and 50.0% vs 61.9%, *P* = .562).

**Table 3:** Cumulative improvement rates of specific endometriosis symptoms in 24 months.

	Group A		Group B		Group A vs Group B		
Timeline	n/N	% (95%CI)	n/N	% (95%CI)	Odds ratio	95% CI	*P*-value
Pain symptoms							
3-mo	114/144	79.2 (71.6, 85.5)	185/216	85.6 (80.3, 90.0)	0.5	(-0.1, 1.0)	.110
6-mo	116/144	80.6 (73.1, 86.7)	194/216	89.8 (85.0, 93.5)	0.8	(0.2, 1.4)	.014
9-mo	118/144	81.9 (74.7, 87.9)	196/216	90.7 (86.1, 94.3)	0.8	(0.1, 1.4)	.016
12-mo	119/144	82.6 (75.4, 88.4)	196/216	90.7 (86.1, 94.3)	0.8	(0.1, 1.4)	.025
18-mo	119/144	82.6 (75.4, 88.4)	196/216	90.7 (86.1, 94.3)	0.8	(0.1, 1.4)	.025
24-mo ^α^	119/144	82.6 (75.4, 88.4)	196/216	90.7 (86.1, 94.3)	0.8	(0.1, 1.4)	.025
Menstrual disorders							
3-mo	20/31	64.5 (45.4.80.8)	31/42	73.8 (58.0, 86.1)	0.4	(-0.6, 1.4)	.394
6-mo	20/31	64.5 (45.4.80.8)	33/42	78.6 (63.2, 89.7)	0.7	(-0.3, 1.7)	.187
9-mo	20/31	64.5 (45.4.80.8)	33/42	78.6 (63.2, 89.7)	0.7	(-0.3, 1.7)	.187
12-mo	20/31	64.5 (45.4.80.8)	33/42	78.6 (63.2, 89.7)	0.7	(-0.3, 1.7)	.187
18-mo	20/31	64.5 (45.4.80.8)	33/42	78.6 (63.2, 89.7)	0.7	(-0.3, 1.7)	.187
24-mo^α^	20/31	64.5 (45.4, 80.8)	33/42	78.6 (63.2,89.7)	0.7	(-0.3, 1.7)	.187
Gastro-intestinal symptoms							
3-mo	25/41	61.0 (44.5, 75.8)	61/78	78.2 (67.4, 86.8)	0.8	(0.0, 1.7)	.049
6-mo	25/41	61.0 (44.5, 75.8)	63/78	80.8 (70.3, 88.8)	1.0	(0.1, 1.8)	.022
9-mo	25/41	61.0 (44.5, 75.8)	65/78	83.3 (73.2, 90.8)	1.2	(0.3, 2.0)	.008
12-mo	25/41	61.0 (44.5, 75.8)	65/78	83.3 (73.2, 90.8)	1.2	(0.3, 2.0)	.008
18-mo	25/41	61.0 (44.5, 75.8)	65/78	83.3 (73.2, 90.8)	1.2	(0.3, 2.0)	.008
24-mo^α^	25/41	61.0 (44.5, 75.8)	65/78	83.3 (73.2, 90.8)	1.2	(0.3, 2.0)	.008
Urinary symptoms							
3-mo	4/8	50.0 (15.7, 84.3)	13/21	61.9 (38.4, 81.9)	0.5	(-1.2,2.1)	.562
6-mo	4/8	50.0 (15.7, 84.3)	13/21	61.9 (38.4, 81.9)	0.5	(-1.2,2.1)	.562
9-mo	4/8	50.0 (15.7, 84.3)	13/21	61.9 (38.4, 81.9)	0.5	(-1.2,2.1)	.562
12-mo	4/8	50.0 (15.7, 84.3)	13/21	61.9 (38.4, 81.9)	0.5	(-1.2,2.1)	.562
18-mo	4/8	50.0 (15.7, 84.3)	13/21	61.9 (38.4, 81.9)	0.5	(-1.2,2.1)	.562
24-mo^α^	4/8	50.0 (15.7, 84.3)	13/21	61.9 (38.4, 81.9)	0.5	(-1.2,2.1)	.562

The majority (60.0%) of pain recurrence for group A occurred during 6–9 months of the treatment while the majority of recurrence (53.5%) in group B occurred during 12–24 months. Table 4 describes the recurrence rate of pain during 24-months. For study population, the 12-months and 24-months cumulative recurrence rates (11.3% vs 13.8% and 16.1% vs 26.0%) of specific symptoms in group A were lower than those in group B, albeit not statistically significant (*P* = .568 and *P* = .094). However, for patients with null or mild symptoms at 3-month, the 12-months and 24-months cumulative recurrence rates (11.9% vs 11.9% and 15.6% vs 23.8%) of symptoms between two groups were not statistically significant yet (*P* = .990 and *P* = .145).15/148 and 43/226 patients had pain after primary symptoms disappearance in group A and group B respectively; whereas, 16/157 and 35/191 patients with null or mild symptoms at 3-months in both groups experienced pain respectively. The pain-free interval for study population between the two groups (see Figure S1, Supplemental Digital Content, Supplementary content, which shows the time to relapse of pain) was not statistically significant (*P* = .072). And patients with null or mild symptoms at 3-months between the two groups was not statistically significant yet (*P* = .160).

**Table 4:** Cumulative recurrence rates of specific endometriosis symptoms in 24 months.

	Group A		Group B		Group A vs Group B ^α^		
Timeline	n/N	% (95%CI)	n/N	% (95%CI)	Odds ratio	95% CI	*P*-value
Study population							
12-mo	14/124	11.3 (6.3, 18.2)	27/196	13.8 (9.3, 19.4)	0.2	(-0.4, 0.9)	.568
24-mo	20/124	16.1 (10.1, 23.8)	51/196	26.0 (20.0, 32.8)	0.6	(-0.1, 1.2)	.094
Patients with null or mild pain symptoms at 3-mo							
12-mo	16/135	11.9 (6.9, 18.5)	20/168	11.9 (7.4, 17.8)	0.0	(-0.7, 0.7)	.990
24-mo	21/135	15.6 (9.9, 22.8)	40/168	23.8 (17.6, 31.0)	0.5	(-0.1, 1.1)	.145

The hazard ratio (HR) of multivariate Cox regression analysis (see Table S1, Supplemental Digital Content, Supplementary content, which illustrates the predictive factors of triptorelin therapy duration) suggested that triptorelin treatment duration was longer for elder patients than youngers at surgery (HR 0.983; 95% confidence interval [CI]: 0.968 - 1.000; *P* = .044) and also for patients who previously received hormonal treatment for endometriosis than those who did not (HR 0.724; 95% CI:0.543 - 0.950; *P* = .024), and shorter in patients who were infertile versus fertile (HR 1.401; 95% CI:1.046 - 1.844; *P* = .019).

## 4 Discussion

To our knowledge, this is the first prospective multicentre study to evaluate symptom control by comparing postoperative triptorelin treatment duration among Chinese patients with DIE.

The results of this study suggest that triptorelin significantly decreases pain, menstrual disorders, gastro-intestinal symptom scores irrespective of treatment duration. Pain symptoms score at 6-months in group B with 4 to 6 triptorelin injections was significantly lower than those in group A with 1 to 3 injections, while the 24-months scores were with no statistical significance between two groups. It illustrates that treatment with triptorelin following conservative surgery may associated with a decrease in the intensity of pain symptoms in the short-term, and the long-term effects of shorter or longer triptorelin therapy may depend on long-term clinical care. A published study performed in China using 4 injections (6-week per cycle for 24 weeks) versus 6 injections (4-week per cycle for 24 weeks) of triptorelin depot regimen in the treatment of patients with adenomyosis and endometriosis, which suggested that both therapeutic regimens achieved similar efficacy on decreasing dysmenorrhoea score and similar symptom recurrence.^[19]^ The results were also in accordance with another study by Liu et al published in China.^[20]^

The 6-month and 24-month cumulative improvement rates of pain and gastro-intestinal symptoms were significantly higher in group B than those in group A in the study. Moreover, the improvement rate of pain symptoms in group B was significantly higher at 6-month than that in group A. Likewise, the 18-month and 24-month rates of gastro-intestinal symptoms in group B were significantly higher than group A. The results illustrate that longer treatment with triptorelin following conservative surgery may be associated with greater improvement of pain symptoms in the short-term and greater improvement of gastro-intestinal symptoms in the long-term.

The rates of symptom recurrence were slightly lower in group A than those in group B between 10% and 25% in 12 to 24 months which were consistent with the recurrence rate of 21% reported in previous study,^[21]^ even if comparison between studies has limitations due to different settings and varying designs. For patients with null or mild pain symptoms at-3month, continued use of drugs may slightly decrease the rate of recurrence. At baseline, the number of patients with mild symptoms in group A was more than those in group B, which might be the reason for the slightly higher recurrence rate in group B. In our study, 60.0% of patients in group A experienced pain recurrence within 6 to 9 months follow-up while 53.5% in group B occurred within 12 to 24 months. Furthermore, pain-free intervals for group A were shorter than those for group B in total population and patients with null or mild symptoms at 3-months. It reveals that longer triptorelin treatment duration might lengthen pain-free interval within 24 months. Moreover, published study proved that proper lifestyle, diet rich in vegetables, omega-3 polyunsaturated fatty acids with less consumption of red meat, coffee, alcohol and trans fats play an important role in whole therapy.^[22]^ And published study showed that proper lifestyle, intakes of magnesium, phosphorus, calcium, and vitamin D lower the risk of occurrence and strengthen the effectiveness of treatment of endometriosis.^[23]^

In our study, patients with longer triptorelin treatment duration were those who were fertile, older at surgery, previously with hormonal treatment for endometriosis. In clinical practice, the treatment duration might be concerned with patients health status, pregnancy history, the family economic revenue, history of treatment, severity of disease, and sensitivity to triptorelin injections.

The strength of this study is that it is the first study on symptom control in comparison of different duration of post-operative triptorelin therapy in patients with DIE conducted in multi-centres in China. The study suggests that the treatment duration of triptorelin following conservative surgery may affect the efficacy. Therefore, exploring predictive factors of increasing treatment duration was meaningful. A few limitations of this study must be acknowledged. The main limitation of the study may be that the baseline characteristics between two groups were not balanced and the propensity score matching method was not conducted in the study. In addition, data missing in the non-interventional observation study was inevitable. However, the observational study was performed in the real-world clinical practice, it provides guidance for clinical pharmacy. Additionally, the study was finished from multi-centres, diagnostic and therapeutic capabilities may vary from hospitals, so the central effect was considered by the post-hoc analysis. Moreover, that was an exploratory analysis with a multiplicity of test without adjustment.

## 5 Conclusions

In women with DIE, longer treatment with triptorelin following conservative surgery was associated with a decrease in the intensity of pain symptoms in the short-term, greater improvement of pain symptoms in the short-term and greater improvement of gastro-intestinal symptoms in the long-term, but a higher rate of recurrence. Moreover, it might lengthen the pain-free interval. Age at surgery, previously hormonal treatment for endometriosis and infertility are the predictive factors of increased triptorelin treatment duration.

## Acknowledgments

The authors thank all patients involved in the study, as well as their caregivers, care team, investigators and research staff in participating institutions. The authors also thank Beijing Preintell Biomed Co., Ltd, who provided medical writing support, which was sponsored by Ipsen, in accordance with Good Publication Practice guidelines. Furthermore, the authors gratefully acknowledge all the investigators of research institutions for their contribution to the study: Zheng Guan (Chinese PLA General Hospital), Yan Huang (The Second Affiliated Hospital of Army Medical University), Liguo Ma (Shenzhen People's Hospital), Jian Zhang (International Peace Maternity and Child Health Hospital of the China Welfare Institute), Ouping Huang (Jiangxi Maternal and Child Health Hospital), Hongbo Wang (Wuhan Union Hospital of Tongji Medical College of Huazhong University of Science and Technology), Dazhen Yang (Nanjing Maternity and Child Health Care Hospital), Dong Zhao (The First Maternal and Infant Hospital of Shanghai), Weidong Zhao (Anhui Provincial Cancer Hospital), Zhiqing Liang (Southwest Hospital of the Third Military Medical University), Lifang Sun (The Fourth Medical College of Peking University and Jishuitan Orthopaedic College of Tsinghua University).

## Author contributions

WS performed the study and was a major contributor in writing the manuscript. KH, LH, JZ, MH, JW and JZ performed the study and made a contribution to the study design and manuscript draft. VP and HL analysed and interpreted clinical data, provided feedback on the manuscript. XZ designed and performed the study, was a major contributor in writing the manuscript and gave final approval of the version to be submitted. All authors read and approved the final manuscript.

**Conceptualization:** Xinmei Zhang.

**Data curation:** Wenting Sun, Valerie Perrot, Hongbo Li.

**Formal analysis:** Valerie Perrot.

**Funding acquisition:** Xinmei Zhang.

**Investigation:** Wenting Sun, Keqin Hua, Li Hong, Juxin Zhang, Min Hao, Jianliu Wang, Jun Zhang, Xinmei Zhang.

**Methodology:** Keqin Hua, Li Hong, Juxin Zhang, Min Hao, Jianliu Wang, Jun Zhang, Xinmei Zhang.

**Project administration:** Xinmei Zhang.

**Resources:** Keqin Hua, Li Hong, Juxin Zhang, Min Hao, Jianliu Wang, Jun Zhang, Xinmei Zhang.

**Software:** Valerie Perrot.

**Supervision:** Hongbo Li.

**Validation:** Hongbo Li.

**Writing – original draft:** Wenting Sun, Xinmei Zhang.

**Writing – review & editing:** Xinmei Zhang.

## Supplementary Material

Supplemental Digital Content

## Supplementary Material

Supplemental Digital Content

## References

[1] Chapron C Jacob S Dubuisson JB Vieira M Liaras E Fauconnier A. Laparoscopically assisted vaginal management of deep endometriosis infiltrating the rectovaginal septum. Acta Obstet Gynecol Scand 2001;80:349–54. 11264611

[2] Angioni S Pontis A Dessole M Surico D De Cicco Nardone C Melis I. Pain control and quality of life after laparoscopic en-block resection of deep infiltrating endometriosis (DIE) vs. incomplete surgical treatment with or without GnRHa administration after surgery. Arch Gynecol Obstet 2015;291:363–70. 25151027 10.1007/s00404-014-3411-5

[3] Cohen J Ballester M Selleret L. Finding the balance between surgery and medically-assisted reproduction in women with deep infiltrating endometriosis. Minerva Ginecol 2016;68:642–52. 27098393

[4] Koninckx PR Ussia A Adamyan L Wattiez A Donnez J. Deep endometriosis: definition, diagnosis, and treatment. Fertil Steril 2012;98:564–71. 22938769 10.1016/j.fertnstert.2012.07.1061

[5] Szubert M Zietara M Suzin J. Conservative treatment of deep infiltrating endometriosis: review of existing options. Gynecol Endocrinol 2018;34:10–4. 28952821 10.1080/09513590.2017.1381837

[6] Araujo SE Seid VE Marques RM Gomes MT. Advantages of the robotic approach to deep infiltrating rectal endometriosis: because less is more. J Robot Surg 2016;10:165–9. 27072152 10.1007/s11701-016-0586-8

[7] Wilczynski M Wiecka-Plusa M Antosiak B Maciolek-Blewniewska G Majchrzak-Baczmanska D Malinowski A. Rectovaginal endometriosis--analysis of 160 cases. Ginekol Pol 2015;86:896–901. 26995938 10.17772/gp/59274

[8] Hudelist G Aas-Eng MK Birsan T. Pain and fertility outcomes of nerve-sparing, full-thickness disk or segmental bowel resection for deep infiltrating endometriosis-A prospective cohort study. Acta Obstet Gynecol Scand 2018;97:1438–46. 30080244 10.1111/aogs.13436

[9] Uccella S Gisone B Serati M. Functional outcomes of nerve-sparing laparoscopic eradication of deep infiltrating endometriosis: a prospective analysis using validated questionnaires. Arch Gynecol Obstet 2018;298:639–47. 30062386 10.1007/s00404-018-4852-z

[10] Derouich S Attia L Slimani O. Medical treatment of endometriosis. Tunis Med 2015;93:407–12. 26757492

[11] Slopien R Meczekalski B. Aromatase inhibitors in the treatment of endometriosis. Prz Menopauzalny 2016;15:43–7. 27095958 10.5114/pm.2016.58773 PMC4828508

[12] Vercellini P Buggio L Frattaruolo MP Borghi A Dridi D Somigliana E. Medical treatment of endometriosis-related pain. Best Pract Res Clin Obstet Gynaecol 2018;51:68–91. 29530425 10.1016/j.bpobgyn.2018.01.015

[13] Leone Roberti Maggiore U Scala C Remorgida V. Triptorelin for the treatment of endometriosis. Expert Opin Pharmacother 2014;15:1153–79. 24832495 10.1517/14656566.2014.916279

[14] Xue H Liu M Hao W Li Y. Clinical evaluation of laparoscopic surgery combined with triptorelin acetate in patients with endometriosis and infertility. Pak J Med Sci 2018;34:1064–9. 30344551 10.12669/pjms.345.15574 PMC6191787

[15] Zhong YJ Zhang W Zhang WT Cheng J Lu QY Zeng KK. Efficacy and safety of GnRH-a combine with laparoscope conservative surgery in the treatment of the moderate or severe endometriosis. Zhonghua Fu Chan Ke Za Zhi 2013;48:180–2. 23849939

[16] Li Z Zhang HY Zhu YJ Hu YJ Qu PP. A randomized study comparing the side effects and hormonal status of triptorelin and leuprorelin following conservative laparoscopic surgery for ovarian endometriosis in Chinese women. Eur J Obstet Gynecol Reprod Biol 2014;183:164–8. 25461372 10.1016/j.ejogrb.2014.10.022

[17] Choktanasiri W Boonkasemsanti W Sittisomwong T. Long-acting triptorelin for the treatment of endometriosis. Int J Gynaecol Obstet 1996;54:237–43. 8889631 10.1016/0020-7292(96)02698-7

[18] Bergqvist A Bergh T Hogstrom L Mattsson S Nordenskjold F Rasmussen C. Effects of triptorelin versus placebo on the symptoms of endometriosis. Fertil Steril 1998;69:702–8. 9548161 10.1016/s0015-0282(98)00019-3

[19] Kang JL Wang XX Nie ML Huang XH. Efficacy of gonadotropin-releasing hormone agonist and an extended-interval dosing regimen in the treatment of patients with adenomyosis and endometriosis. Gynecol Obstet Invest 2010;69:73–7. 19923847 10.1159/000258683

[20] Liu DY Gu MF Shu JZ Shi YX Wang CY Han ZQ. Efect of triptorelin and an extended-interval dosing regim en in the treatment of patients with endometriosis and adenomyoma. Chin J Obstet Gynecol 2006;41:656–9. 17199917

[21] Loverro G Carriero C Rossi AC Putignano G Nicolardi V Selvaggi L. A randomized study comparing triptorelin or expectant management following conservative laparoscopic surgery for symptomatic stage III-IV endometriosis. Eur J Obstet Gynecol Reprod Biol 2008;136:194–8. 17178185 10.1016/j.ejogrb.2006.10.034

[22] Parazzini F Viganò P Candiani M Fedele L. Diet and endometriosis risk: a literature review. Reproductive BioMedicine Online 2013;26:323–36. 23419794 10.1016/j.rbmo.2012.12.011

[23] Harris HR Chavarro JE Malspeis S. Dairy-food, calcium, magnesium, and vitamin D intake and endometriosis. Am J Epidemiol 2013;177:420–30. 23380045 10.1093/aje/kws247 PMC3626048

