# Supplementary material for: Symptom control after different duration of triptorelin treatment following conservative surgery for deep infiltrating endometriosis: Post-hoc analysis of a multicentre, prospective, real-world study
Source: Medicine (Baltimore). 2021 Jul 30;100(30):e26753. doi: 10.1097/MD.0000000000026753 (PMC8322541; doi:10.1097/MD.0000000000026753)
Supplement: Supplemental Digital Content [file medi-100-e26753-s001.docx]

**Supplemental Digital Content**

**Symptom control after different duration of triptorelin treatment following conservative surgery for deep** **infiltrating endometriosis: post-hoc analysis of a multicentre, prospective, real-world study**

Wenting Sun (MM)^1^, Keqin Hua (PhD)^2^, Li Hong (PhD)^3^, Juxin Zhang (PhD)^4^, Min Hao (PhD)^5^, Jianliu Wang (PhD)^6^, Jun Zhang (PhD)^7^, Valerie Perrot (MD)^8^, Hongbo Li (MM)^9^, and Xinmei Zhang (PhD) ^1*^


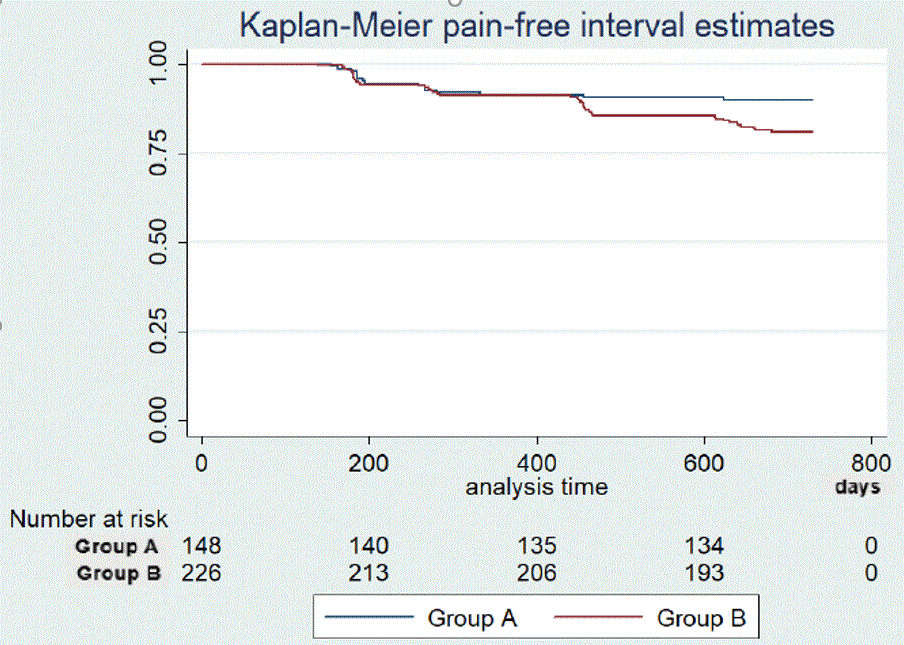


**Figure S1 that show the time to relapse of pain of study population**

Group A: patients received up to 3 injections of triptorelin, NA=156. Group B: patients received 4-6 injections of triptorelin, NB=228.
