# Supplementary material for: Symptom control after different duration of triptorelin treatment following conservative surgery for deep infiltrating endometriosis: Post-hoc analysis of a multicentre, prospective, real-world study
Source: Medicine (Baltimore). 2021 Jul 30;100(30):e26753. doi: 10.1097/MD.0000000000026753 (PMC8322541; doi:10.1097/MD.0000000000026753)
Supplement: Supplemental Digital Content [file medi-100-e26753-s002.docx]

**Supplemental Digital Content**

**Symptom control after different duration of triptorelin treatment following conservative surgery for deep** **infiltrating endometriosis: post-hoc analysis of a multicentre, prospective, real-world study**

Wenting Sun (MM)^1^, Keqin Hua (PhD)^2^, Li Hong (PhD)^3^, Juxin Zhang (PhD)^4^, Min Hao (PhD)^5^, Jianliu Wang (PhD)^6^, Jun Zhang (PhD)^7^, Valerie Perrot (MD)^8^, Hongbo Li (MM)^9^, and Xinmei Zhang (PhD) ^1*^

**Table S1 that illustrates the predictive factors of triptorelin therapy duration of study population** (N=384)

| Parameter | Hazard ratio | 95% CI | p-value |
| --- | --- | --- | --- |
| Age at surgery (years) | 0.983 | (0.968, 1.000) | 0.044 |
| Previously treated with a hormonal treatment for endometriosis | | | |
| No^α^ |  |  |  |
| Yes | 0.724 | (0.543, 0.950) | 0.024 |
| Infertility |  |  |  |
| No^α^ |  |  |  |
| Yes | 1.401 | (1.046, 1.844) | 0.019 |

^α^Reference for level factor. CI: confidence interval.
